# Genotyping Multidrug-Resistant Mycobacterium tuberculosis from Primary Sputum and Decontaminated Sediment with an Integrated Microfluidic Amplification Microarray Test

**DOI:** 10.1128/JCM.01652-17

**Published:** 2018-02-22

**Authors:** Yvonne Linger, Christopher Knickerbocker, David Sipes, Julia Golova, Molly Franke, Roger Calderon, Leonid Lecca, Nitu Thakore, Rebecca Holmberg, Peter Qu, Alexander Kukhtin, Megan B. Murray, Christopher G. Cooney, Darrell P. Chandler

**Affiliations:** a Akonni Biosystems, Inc., Frederick, Maryland, USA; b Harvard Medical School, Department of Global Health and Social Medicine, Boston, Massachusetts, USA; c Socios En Salud Sucursal Perú, Lima, Peru; Carter BloodCare & Baylor University Medical Center

**Keywords:** amplification microarray, closed amplicon, drug resistance, *in vitro* diagnostic, microfluidic, Mycobacterium tuberculosis

## Abstract

There is a growing awareness that molecular diagnostics for detect-to-treat applications will soon need a highly multiplexed mutation detection and identification capability. In this study, we converted an open-amplicon microarray hybridization test for multidrug-resistant (MDR) Mycobacterium tuberculosis into an entirely closed-amplicon consumable (an amplification microarray) and evaluated its performance with matched sputum and sediment extracts. Reproducible genotyping (the limit of detection) was achieved with ∼25 M. tuberculosis genomes (100 fg of M. tuberculosis DNA) per reaction; the estimated shelf life of the test was at least 18 months when it was stored at 4°C. The test detected M. tuberculosis in 99.1% of sputum extracts and 100% of sediment extracts and showed 100% concordance with the results of real-time PCR. The levels of concordance between M. tuberculosis and resistance-associated gene detection were 99.1% and 98.4% for sputum and sediment extracts, respectively. Genotyping results were 100% concordant between sputum and sediment extracts. Relative to the results of culture-based drug susceptibility testing, the test was 97.1% specific and 75.0% sensitive for the detection of rifampin resistance in both sputum and sediment extracts. The specificity for the detection of isoniazid (INH) resistance was 98.4% and 96.8% for sputum and sediment extracts, respectively, and the sensitivity for the detection of INH resistance was 63.6%. The amplification microarray reported the correct genotype for all discordant phenotype/genotype results. On the basis of these data, primary sputum may be considered a preferred specimen for the test. The amplification microarray design, shelf life, and analytical performance metrics are well aligned with consensus product profiles for next-generation drug-resistant M. tuberculosis diagnostics and represent a significant ease-of-use advantage over other hybridization-based tests for diagnosing MDR tuberculosis.

## INTRODUCTION

The global rollout of the Xpert MTB/RIF system unquestionably improved multidrug-resistant (MDR) tuberculosis (TB) case detection and the time to diagnosis but also brought to light several health care, infrastructure, technology, genotyping, and cost considerations for high-priority diagnostics (1–4). At the same time, Mycobacterium tuberculosis whole-genome sequencing efforts involving hundreds or thousands of drug-resistant M. tuberculosis isolates continue to identify new genes and mutations that cause or are correlated with mono-, multi-, or extensive drug resistance or compensate for known mutations (5–12). Host genetic factors are also known to play a role in M. tuberculosis infection and disease (13). Given the genetic complexity and heterogeneous evolution of M. tuberculosis drug resistance (14), whole-genome sequencing rather than biomarker discovery is now being considered for M. tuberculosis diagnostic purposes (15–19). While it is not yet clear if or when whole-genome sequencing will satisfy high-priority M. tuberculosis diagnostic technical product profiles (3), it is evident that the number of genes and mutations necessary to diagnose drug resistance is increasing and that the identification (as opposed to the detection) of single-nucleotide polymorphisms (SNP) will become increasingly important for prescribing a patient-specific treatment regimen that accounts for patient genetics/drug metabolism and minimizes the emergence of new drug-resistant M. tuberculosis phenotypes.

Microarrays were originally invented as a DNA sequencing technology (20–22) and can address the multiple-gene, multiple-mutation challenge of diagnosing drug-resistant TB (23–30). Microarrays have yet to impact clinical practice in the same way as real-time PCR, in part because of poor reproducibility and repeatability, complex work flows (inclusive of sample preparation), user subjectivity, and a host of related technical and nontechnical issues, even in developed countries and centralized testing laboratories (31–34). Considering the clinical user and operating requirements for TB diagnostics (1–3), it becomes clear that microarray (and sequencing) platforms need to be developed from an entirely distinct perspective to harmonize competing user needs and product requirements for MDR-TB and extremely drug resistant (XDR) TB diagnostics.

One way to overcome the inherent complexity of microarray-based diagnostics for routine use (especially in lower-resource settings) is to combine amplification and microarray hybridization within a single microfluidic chamber, confine amplification products within the consumable (i.e., a closed-amplicon device), simplify the total number of biochemical steps necessary to detect mutations, and optimize the assay for use with low-cost, field-portable microarray imagers (35, 36). The objectives of this study were to advance these concepts and develop a closed-amplicon, microarray-based consumable for the detection of MDR-TB, characterize the device's analytical and shelf-life behavior, and evaluate the test on matched primary sputum and *N*-acetyl-l-cysteine (NALC)–NaOH-decontaminated sediment extracts from confirmed or suspected M. tuberculosis-positive patients.

## MATERIALS AND METHODS

### Genomic DNA, isolates, and positive controls.

Purified M. tuberculosis H37Ra DNA was acquired from the American Type Culture Collection (ATCC; Manassas, VA) and quantified on a NanoDrop 3000 fluorometer before use. Materials from the Special Programme for Research and Training in Tropical Diseases (TDR) Tuberculosis Strain Bank (37) (now integrated under BCCM/TIM) were provided as heat-killed crude lysates. Cell lysates were further processed through a BD GeneOhm bead lysis kit (catalogue number 441243; San Diego, CA), and genomic DNA was purified with a Qiagen DNA minikit (catalogue number 51304; Germantown, MD) per the manufacturers' respective instructions, except that samples were incubated at 56°C for 30 min instead of Qiagen's recommended 10 min. Purified nucleic acids were quantified by real-time PCR (see below) relative to the amounts on an external standard curve prepared with M. tuberculosis H37Ra DNA. M13mp18 single-stranded DNA was purchased from New England BioLabs (Ipswich, MA) and diluted to 750 pg ml^−1^ in 10 mM Tris, 1 mM Na_2_ EDTA (pH 7.5). Purified nucleic acids were stored at −20°C until use.

### Primary sputum and decontaminated sediment samples.

This study utilized retrospective, banked sputum and sediment samples that were collected for a different research objective. Samples were originally derived from patients in public-sector clinics in Lima, Peru, if they had symptoms consistent with M. tuberculosis infection and a prior primary sputum sample testing positive for acid-fast bacilli (AFB) by Ziehl-Neelsen staining. No identifying information or additional clinical data were collected from the study participants. All participants completed written informed consent, and the parent study was approved by the Institutional Review Board of the Harvard Medical School and the Ethics Committee of the Peru National Institute of Health. Thereafter, 2 ml of each sputum sample was decontaminated with 2% NaOH and 0.25% NALC for 15 min, neutralized by adding enough saline phosphate buffer to reach a 50-ml total volume, and centrifuged at 3,000 × *g* for 30 min. The sediment was resuspended in 1.5 ml of phosphate-buffered saline, and 0.2 ml of each sample was used to inoculate two Löwenstein-Jensen (LJ) slants. Culture tubes were incubated at 37°C and monitored for growth for up to 8 weeks. Ziehl-Neelsen staining and an M. tuberculosis identification test were performed on the culture-positive sputum specimens to confirm the presence of M. tuberculosis in the primary specimen. All remaining sputum specimens and their paired sediment specimens were stored at −80°C until use in the experiments reported here.

Before use in the amplification microarray experiments reported here, we performed follow-up Ziehl-Neelsen acid-fast staining, some of which generated a smear-negative result for AFB (see Table S1 in the supplemental material), even though the original sample was smear positive for AFB. Clinical samples assigned a scanty AFB smear status upon retesting were considered smear positive for this purpose of this study.

Standard drug susceptibility tests (DSTs) were retrospectively performed on positive LJ cultures that were still viable at the time that this study was initiated.

### Automated DNA extraction from sputum and sediment.

Total genomic DNA was extracted from the primary sputum and NALC-NaOH-decontaminated sediment using an Akonni TruTip automated workstation, 1.2 ml SPT TruTips, and preloaded reagent plates. Briefly, 500 μl primary sputum was mixed with 80 μl Akonni liquefaction buffer, and the mixture was incubated at 56°C for 20 min with intermittent mixing. Thereafter, a batch of seven sputum or sediment samples and one water blank (500 μl) were loaded onto the workstation and processed in parallel with an automated protocol consisting of 10 min of magnetically induced vortexing, a 10-min incubation at 56°C, total nucleic acid binding to the matrix, washing and drying, and DNA elution in 100 μl 10 mM Tris-HCl (pH 8.0). Purified DNA was stored at −20°C until use.

### IS*6110*-specific quantitative PCR.

M. tuberculosis-specific DNA in nucleic acid extracts was amplified by real-time PCR using a Roche LightCycler 480 instrument and the IS*6110* insertion element as a proxy for M. tuberculosis in the primary specimen (38). Briefly, 5 μl of each nucleic acid extract was combined with 20 μl master mix in a 96-well plate to achieve a final reaction composition of 1× LightCycler FastStart DNA Master HybProbe buffer and enzyme (Roche), 2.5 mM MgCl_2_, 0.45 μM forward primer (5′-GGG-TAG-CAG-ACC-TCA-CCT-ATG), 1.35 μM reverse primer (5′-AGC-GTA-GGC-GTC-GGT-GA), and 25 nM minor groove binding internal probe (5′ 6FAM-TCG-CCT-ACG-TGG-CCT-TT-MGB, where 6FAM is 6-carboxyfluorescein). The microtiter plates were loaded onto the thermal cycler, denatured for 10 min at 95°C, and cycled for 45 cycles of 95°C for 15 s and 60°C for 60 s.

### Phenotypic drug susceptibility testing.

M. tuberculosis isolates recovered from positive LJ slants were tested for drug susceptibility using a Bactec MGIT 960 system (Becton, Dickinson, Sparks, MD) according to the manufacturer's instructions. The final antibiotic concentrations in MGIT tubes were 0.1 and 1.0 μg ml^−1^ for isoniazid (INH) and rifampin (RIF), respectively.

### Microfluidic amplification microarray primers, probes, and synthetic DNA standards.

Microarray primers and probes were designed against M. tuberculosis mutations known to confer an RIF and INH resistance phenotype (Table 1). Five PCR primer pairs were designed to work together in a multiplex, asymmetric master mix. One of the primers in each pair was synthesized with a Cy3 label and incorporated into the multiplex reaction mixture at 5 to 10 times the concentration of the unlabeled primer. The PCR primer and microarray probe sequences are identical to those of the PCR primers and microarray probes used in a previous study (36). PCR primers were synthesized by the use of standard phosphoramidite chemistry at Akonni Biosystems, purified by high-performance liquid chromatography (HPLC), and quantified by UV absorption before use. The resulting Cy3-labeled amplicons ranged from 92 to 139 nucleotides in length.

**TABLE 1:** Integrated microfluidic amplification microarray genetic coverage^*a*^

Drug	Gene	Amplicon size (nt)	Targeted mutation, target, or description
RIF	*rpoB*	139	507DEL, Q510H, L511P, L511R, S512T, S512R, Q513L, Q513K, Q513P, M515I, D516E, D516Y, D516G, D516V, S522L, L524S, H526D, H526R, H526L, H526Q (CAA), H526Q (CAG), H526C, H526N, H526P, H526Y, S531W, S531L, S531Q, S531C, L533P
INH	*katG*	127	S315T (ACC), S315T (ACA), S315N
INH	*inhA* promoter	106	−8A, −8C, −15T, −17T
NA	IS*6110*	99	M. tuberculosis complex
NA	M13	92	Internal positive control

a nt, number of nucleotides; DEL, deletion; NA, not applicable.

Microarray probes were synthesized by Akonni with a custom 3′ linker and purified to reach >90% purity by HPLC. Probe purity was measured and confirmed by electrospray ionization mass spectrometry. Microarrays contained at least one universal hybridization probe for each resistance-associated gene and primer pair to verify that M. tuberculosis gene targets were amplified from each sample. At least one matched pair of microarray probes (wild type [WT] and single-nucleotide mutant [MU]) was included for each mutation of interest. Control probes included a Cy3 beacon for manufacturing quality control and positional reference, a probe for an M13 internal positive amplification and inhibition control (internal positive control [IPC]), and control probes for *rpoB*, *katG*, and *inhA* amplification and detection.

### Amplification microarray manufacture and quality control.

Gel element arrays were manufactured on custom-coated glass substrates using a 4% copolymer, essentially as described in reference 39. Cy3 fiducial markers were resuspended in the gel precursor at 1 μM, and all other probes were resuspended at a 50 μM concentration before printing. The photopolymerized, washed, and dried microarrays were stored at 4°C for up to 1 week until assembly. All microarrays were visually inspected for gel element presence, a uniform 3-dimensional morphology, and Cy3 beacon fluorescence/uniformity (acceptance criteria, a coefficient of variation of <4% within and between arrays in a production run) before use.

The microarray consumable design is substantially similar to that described elsewhere (35, 40). Essential features of the microfluidic design are that target amplification and microarray hybridization occur simultaneously within a single microfluidic chamber (75 μl), and all amplified products and wash solutions are retained within an integrated waste chamber during and after the wash step (i.e., a closed-amplicon consumable). Microfluidic spacers and cover films were precut, aligned to the microarray substrate with custom jigs, and loosely joined with a pressure roller before permanent lamination in a controlled press. Preassembled waste chambers, inlet ports, and inlet port cover seals were then aligned and loosely affixed before permanent lamination. All finished assemblies were visually inspected for gel element array damage, gross structural defects, adhesive or plastic debris, fluorescent particles that might interfere with automated microarray image analysis, and uniform Cy3 beacon fluorescence (as described above). Integrated consumables that passed final inspection were stored in a vacuum-sealed slide box at 4°C until use (for <2 months, except in the shelf-life study described below).

### Amplification microarray shelf life.

Replicate amplification microarray consumables were manufactured over the course of 2 days, individually wrapped, placed inside vacuum-sealed boxes, and stored at 4°C. At 1, 3, 6, and 18 months, replicate amplification microarrays were removed from storage and processed with 10 pg M. tuberculosis H37Ra genomic DNA per reaction mixture, as described below.

### Amplification, hybridization, washing, and detection.

Purified nucleic acid extract (approximately 21 μl) was combined with the PCR master mix to achieve an 80-μl total reaction volume consisting of 1× Qiagen HotStar *Taq* Plus buffer and enzyme, 7.6% formamide, 5% dimethyl sulfoxide, 1 mg ml^−1^ nonacetylated bovine serum albumin, 4 units of additional HotStar *Taq* (Qiagen), 750 fg internal positive control, and each primer at a final concentration of 0.04 to 1.2 μM. Seventy-five microliters of each reaction mixture was loaded into an amplification microarray, and the inlet port was sealed with a pierceable foil cover. Thereafter, the amplification microarrays were placed on a Quanta (Hain Life Science, UK) QB-96 flat block thermal cycler and subjected to a touchdown thermal cycling program consisting of an initial denaturation for 5 min at 89°C; 30 cycles of 89°C for 45 s, 60 to 55°C (touchdown) for 1 min, and 65°C for 30 s; 20 cycles of 89°C for 45 s, 55°C for 1 min, and 65°C for 30 s; a final extension at 65°C for 3 min; and a postamplification hybridization at 55°C for 3 h. An external positive control (10 pg purified genomic DNA of known genotype) and negative control (water blank) were run with each batch of amplification microarrays.

After amplification and hybridization, the amplification microarrays were washed by piercing the foil seal with a 1-ml pipette tip and flushing 1 ml 1× SSPE (1× SSPE is 0.18 M NaCl, 10 mM NaH_2_PO_4_, and 1 mM EDTA [pH 7.7])–0.01% Triton X-100 through the reaction chamber in a single bolus. The microfluidic design is such that the wash buffer imbibes into the waste chamber, effectively rendering the microarray chamber dry and ready for immediate imaging.

### Automated image and data analysis.

Washed and intact amplification microarray consumables were imaged for 0.2 s on a prototype Akonni Dx2000 imager consisting of a high-intensity green light-emitting diode (LED), custom optics, a noncooled charge-coupled-device camera, and Akonni automated gridding, segmentation, and data analysis software. An integrated signal intensity and a local background signal were acquired for each gel element on the array. The standard deviation of each local background was calculated, and then an average was taken for all local backgrounds. Noise was then calculated as 3 · (average standard deviation for all local backgrounds) · 2 · *R*_int_, where *R*_int_ is the radius of the fixed circle cell used to acquire the signal. A test was declared valid if the internal positive-control probe generated signal-to-noise (SNR) values of ≥3 or the IS*6110* target was detected at an SNR value of ≥10. Otherwise, the test was deemed invalid and the test results were not reported. If IS*6110* was detected at an SNR value of ≥10, then the outputs from the internal control probes were reported as not applicable. Deferring the interpretation of internal control probes in the event of an “M. tuberculosis detected” result is based on the fact that the internal positive control is included at a concentration very near its limit of detection (LoD). In those cases where there is abundant M. tuberculosis DNA in the asymmetric PCR, there may be preferential amplification of the M. tuberculosis genes such that IPC amplification is limited and the IPC SNR value is <3 (i.e., it is not detected).

Positive detection of the IS*6110* target at an SNR value of ≥10 triggered the automated analysis of universal *rpoB*, *katG*, and *inhA* probes. For a universal probe(s) with an SNR value of <3, the susceptibility or resistance report for the associated drug was deemed indeterminate and there was no further analysis of wild-type or mutant probe signals. Otherwise, universal probe SNR values of ≥3 triggered the analysis of wild-type and mutant probe discrimination ratios (*D*). For the *rpoB* gene, both universal probes needed an SNR value of ≥3 to advance the analysis. If at least one of the two paired (wild-type or mutant) probes had an integrated signal intensity greater than the noise floor (defined above), then *D* = (SNR_WT_ − SNR_MU_)/(SNR_WT_ + SNR_MU_), where SNR_WT_ is the SNR value for the wild type and SNR_MU_ is the SNR value for the mutant. Otherwise, *D* was not calculated and the output for the specific mutation was deemed indeterminate. Any value of *D* of <0 was reported as a mutation at the targeted nucleotide position with a “resistance detected” output and an itemized list of the associated mutation(s) from Table 1. If all gene-specific probes generated *D* values of ≥0, then the gene was reported to be the wild type and led to a “resistance not detected” output for the associated drug. Thus, the algorithm and software report on mono- or multidrug resistance. Near the limits of detection and depending on multiplex amplification efficiency, the software may also report susceptibility or resistance for only one antibiotic (RIF or INH), while the drug resistance profile for the other antibiotic is reported as “indeterminate.”

### Discrepant samples.

For those samples in which there was a difference between the results of phenotypic DST and amplification microarray genotyping, the corresponding *rpoB*, *katG*, or *inhA* gene (*n* = 26 total reactions) was amplified from the corresponding extract and subjected to bidirectional DNA sequencing (Eurofins MWG Operon, Louisville, KY).

## RESULTS

### Clinical sample characteristics.

There were 146 sputum samples available for this study. Those samples that generated a contaminated culture were excluded from further analysis, and the results for the samples are not reported here. The remaining 130 samples were categorized by smear and culture status, and the results are shown in Table 2. One sputum sample was consumed in its entirety during NALC-NaOH decontamination, resulting in 129 paired sputum and sediment samples plus one unpaired sediment sample (289 total amplification microarray tests). RIF and INH phenotypic drug susceptibility data were available for only 74 of the 114 culture-positive clinical specimens. Complete smear, culture, DST, and amplification microarray data are itemized in Table S1 in the supplemental material.

**TABLE 2:** Amplification microarray M. tuberculosis and resistance-associated gene detection rate

Sample^*b*^	Target	Detection rate (%)			
		Smear-positive specimens		Smear-negative specimens	
		Culture-positive specimens (*n* = 107 or 108^*a*^)	Culture-negative specimens (*n* = 8)	Culture-positive specimens (*n* = 5)	Culture-negative specimens (*n* = 9)
Sputum extracts	M. tuberculosis	100	100	80	89
	*rpoB*	100	100	100	100
	*katG*	100	88	100	100
	*inhA*	100	100	100	100
Sediment extracts	M. tuberculosis	100	100	100	67
	*rpoB*	99	100	100	83
	*katG*	100	100	100	80
	*inhA*	100	100	80	100

a There were 107 and 108 smear- and culture-positive sputum and sediment extracts, respectively.

b Resistance-associated genes were scored only if M. tuberculosis (the IS*6110* element) was detected by the amplification microarray.

### Analytical specificity and limits of detection.

Microarray probe specificity against clinical M. tuberculosis isolates was largely established in prior work (36), and DNAs from 25 well-characterized (i.e., sequenced) M. tuberculosis isolates were again used here as external positive controls throughout the study (*n* = 28 batch runs). All positive and negative (reagent blank) controls behaved as expected, with no false-positive or false-negative results and correct SNP genotyping and identification.

**FIG 1:**
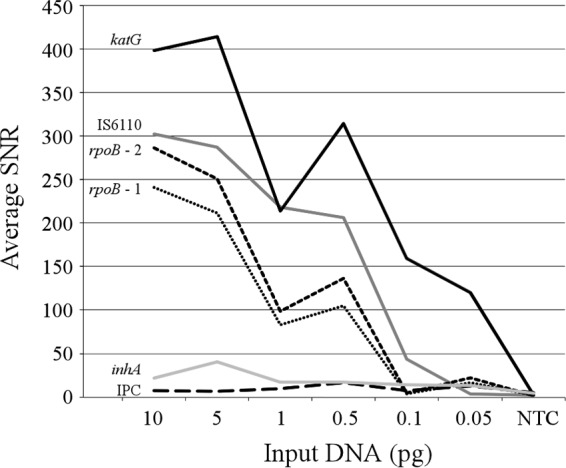
Analytical LoD. IPC, internal positive control; NTC, no-template control.

Analytical limits of detection for the test were estimated by analyzing triplicate dilutions of wild-type M. tuberculosis H37Ra genomic DNA. Average signal-to-noise ratios (SNR values) for the three replicates are plotted in Fig. 1 for the internal positive controls (IPCs) and universal, resistance-associated gene probes. All tests were valid, and the IS*6110* marker was detected at an SNR value of >10 for all dilutions and replicates down to and including 100 fg of genomic DNA. At 50 fg DNA input, one IS*6110* signal did not exceed the SNR value threshold for positive detection, which, according to the preestablished decision logic, terminated the analysis of other probe signals. Positive amplification and positive detection were achieved for the universal, resistance-associated gene probes for all replicates down to and including the 100-fg DNA input, but the *rpoB* probes were undetectable in one replicate at 50 fg DNA. On the basis of the SNR values, the *katG* gene appeared to be preferentially amplified over all other targets and had an average SNR value of 120 even with the 50-fg DNA input, perhaps to the detriment of *inhA* amplification. Correct genotyping and the correct drug susceptibility determination were achieved for all replicates where the test was valid and the universal, resistance-associated gene probes were detected at an SNR value of ≥3, including those replicates at 50 fg that met all predetermined decision logic criteria. We therefore estimated the analytical limit of detection to be ∼100 fg M. tuberculosis genomic DNA per reaction, or ∼25 cell equivalents, assuming 4.4 × 10^6^ bp (41) and ∼4 fg genomic DNA per cell.

### Amplification microarray shelf life.

**FIG 2:**
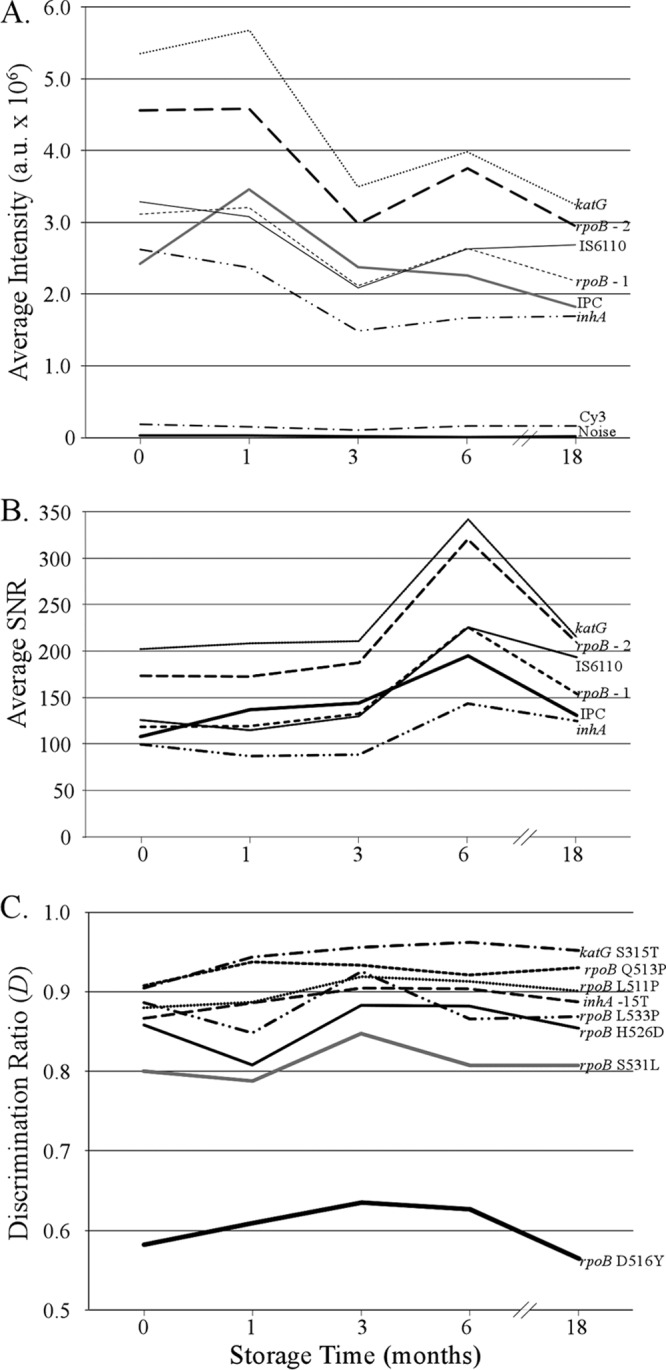
Average fluorescent intensity (A), signal-to-noise ratios (B), and discrimination ratios (C) after amplification microarray storage at 4°C. a.u., absorbance units; IPC, internal positive control; Cy3, fluorescently labeled fiducial marker.

The average, integrated signal intensities, SNR values, and discrimination ratios (*D*) for internal positive controls, universal resistance-associated gene probes, and selected mutations are shown in Fig. 2. After an initial 30 to 40% decrease in average intensity, all probe responses were stable to 18 months of storage. There was no appreciable increase in background noise, genotyping results were correct for all tests, and the behavior of all other probes on the array was substantially similar to that shown in Fig. 2 (not shown).

### Nucleic acid recovery and M. tuberculosis detection.

Real-time PCR detected the IS*6110* element in all samples (average threshold cycle [*C_T_*] value, ≤37) except for one sputum extract and two sediment extracts that were smear negative and culture negative (S−/C−). Two primary sputum samples and 8 sediment extracts resulted in an IS*6110 C_T_* value of >35, and all but one of these samples occurred among smear-negative samples. These results reflect the presence of a relatively low concentration of M. tuberculosis in the original sample. Excluding S−/C− samples, the automated sample preparation recovered significantly more M. tuberculosis DNA from the primary sputum than from the matched sediments (average *C_T_* = 22.6 versus 25.2; *P* ≪ 0.0001).

### M. tuberculosis and gene detection efficacy.

M. tuberculosis detection efficacy relative to smear and culture status is summarized in Table 2. Excluding the nine S−/C− samples for which the true state of M. tuberculosis infection could not be determined, the amplification microarray detected M. tuberculosis in 99.1% (119/120) of sputum extracts and 100% (127/127) of sediment extracts. There was one failure to detect M. tuberculosis in a culture-positive sputum sample (Table S1, sample 91), but the sample was also M. tuberculosis negative by real-time PCR. The results of real-time PCR and the amplification microarray were 99.1% concordant for M. tuberculosis detection (229/231 amplification reactions), with the single discrepancy occurring in an S−/C− sample (Table S1, sample 116). The concordance between amplification microarray M. tuberculosis and resistance-associated gene detection was 99.1% (119/120) and 98.4% (125/127) for sputum and sediment extracts, respectively. There were no cases where a gene probe was detected in the absence of an IS*6110* signal, and a failure to detect the resistance-associated genes was always associated with low M. tuberculosis DNA quantities in the extract (Table S1).

### Amplification microarray genotyping relative to DST.

Of the 129 paired sputum and sediment extracts, the amplification microarray test identified the isolates in 6 to be INH monoresistant, the isolates in 4 to be RIF monoresistant, and the isolates in 14 to be MDR. Of the 74 culture-based DST results, 8 indicated that the isolates were INH monoresistant, 1 indicated that the isolate was RIF monoresistant, and 3 indicated that the isolates were MDR. Relative to the results of the culture-based DST, the amplification microarray test was 97.1% specific and 75.0% sensitive for the detection of RIF resistance in the isolates in both sputum and sediment extracts. The specificity for the detection of INH resistance was 98.4% and 96.8% for sputum and sediment extracts, respectively, and the sensitivity for the detection of INH resistance was 63.6%. Amplification microarray genotyping results were 100% concordant between sputum and sediment extracts for the subset of 74 samples for which a phenotypic DST result was available. Any DST and amplification microarray genotyping outcomes were resolved by bidirectional DNA sequencing rather than repeat DST because of concerns about the integrity of the isolates (which were not banked or frozen) and the age of the banked sputum and sediment samples. In all cases, the microarray genotype matched the corresponding DNA sequence.

Relative to the results of DST, there were three false-resistant (FR) genotype calls for RIF and INH resistance (Table S1, samples 34, 106, and 109). One of those results (the sample 109 sediment extract) could be explained by poor DNA recovery relative to that for its paired sputum specimen, with the concentration of available DNA being at or near the LoD of the test (*C_T_* = 36.0). Otherwise, the FR results may be explained by a failure of the MGIT DST system to detect antibiotic resistance (42). There was one RIF-false-susceptible result from a smear-negative, culture-positive sample. Again, the microarray signals were strong, the sediment and sputum extracts generated the same genotype, and the amplification microarray genotypes matched the corresponding DNA sequence. The RIF-false-susceptible genotype could therefore represent RIF resistance arising from a mutation elsewhere in the genome (9, 10, 14, 43). The four INH-false-susceptible results, all of which were correct from the perspective of the DNA sequence, are most likely a consequence of limited gene and SNP coverage on the amplification microarray relative to the number of genes and SNPs now linked to INH resistance (5–10).

### Genotyping in sputum versus sediments.

Excluding the S−/C− extracts, the genotyping results from the sputum and sediment extracts were 99.1% concordant (119/120 samples). The single discrepancy (Table S1, sample 109) was a culture-positive sample with a smear result of +1, where the results of genotyping for RIF and INH resistance for the sediment (*C_T_* = 36.0) were indeterminate, whereas the matched sputum extract (*C_T_* = 24.7) reported RIF and INH susceptibility. This result may be explained by the loss of M. tuberculosis cells during NALC-NaOH decontamination and sedimentation and, subsequently, poor DNA recovery from the sediment.

## DISCUSSION

### Amplification microarrays.

The genetic complexity of M. tuberculosis drug resistance and the host response indicate that next-generation M. tuberculosis diagnostics will require relatively high levels of multiplexing and an ability to specifically identify resistance-conferring or compensatory mutations that are present in the isolates in a specimen. For this reason, microarrays remain a potentially useful platform for M. tuberculosis diagnostics and personalized medicine, provided that they can be configured to meet known, high-priority product requirements. In this context, the premise of an amplification microarray is to simplify microarray-based biochemistry and the work flow for clinical practice, ideally (and eventually) near the point of use. In this study, we converted a previously described open-amplicon MDR-TB microarray test (36) into an entirely closed-amplicon consumable, a work-flow transition that is conceptually similar to the conversion of PCR into real-time PCR. The simplifying microfluidic principle(s) includes geometries and materials to pin the contact line of the liquid meniscus to confine the amplification reagent mixture to the gel element array amplification chamber during thermal cycling. A closed-amplicon consumable is maintained by using a hydrophilic absorbent to help imbibe all liquids into the waste chamber during the wash step without the need for active pumps or valves. Relative to other hybridization-based tests for the detection of drug-resistant M. tuberculosis (24–27, 30, 44–47), the amplification microarray described here combines up to seven manual steps and processes into a single step in a single microfluidic reaction chamber. It is also important to note that the resulting amplification microarray differs from seemingly related PCR array technologies in that the amplification microarray is based on a homogeneous multiplexed reaction, whereas PCR arrays split the sample into multiple reaction wells, droplets, or channels. Sample splitting can become limiting when the diagnostic objective requires a very stringent limit of detection for many targets and SNPs, as is the case for drug-resistant M. tuberculosis.

In addition to the data in Fig. 2, anecdotal evidence from numerous shipments and collaborative efforts (not shown) further indicates that there is no degradation of the amplification microarray signals or performance when the amplification microarray is shipped at ambient temperature by air freight, including when the amplification microarray is shipped overseas and subjected to multiweek, uncontrolled storage while shipments pass through customs. Taken together, the amplification microarray microfluidic design and shelf-life data show that the amplification microarray represents a significant integration step toward aligning microarray technology with high-priority M. tuberculosis target product profiles (3).

### Amplification microarray performance.

The amplification microarray analytical specificity against 25 external positive controls was consistent with that found in prior work, and the lower limit of detection and reproducible genotyping (25 cell equivalents of genomic DNA per reaction) represent a fourfold improvement over those of the predecessor (open-amplicon) test described elsewhere (36). The analytical sensitivity is therefore on par with that of real-time PCR or isothermal amplification technologies, a conclusion further supported by the 99.1% concordance rate between real-time PCR and amplification microarray detection of the IS*6110* element. Given that 20% of the nucleic acid extract was included in each amplification reaction, the implication is that TruTip (or other sample preparation technology) needs to generate only approximately 0.5 pg M. tuberculosis DNA per extraction (and elution into 100 μl) to generate a reproducible MDR M. tuberculosis genotyping result.

That the automated workstation recovered significantly more M. tuberculosis DNA from primary sputum than from decontaminated sediments is significant in itself, as the consensus opinion is that primary sputum is the preferred (or required) sample type for new M. tuberculosis molecular diagnostics (3). While the total number of smear-negative samples in this study was purposely low, the data also suggest that the combined sample preparation and amplification microarray method has the analytical sensitivity needed to detect M. tuberculosis and the drug resistance genotype in smear-negative sputum specimens. Confirmation of this hypothesis will require a new study with an expanded set of smear-negative samples. In the interim, the analytical performance data suggest that the method can meet the diagnostic sensitivity requirements for patient triage (or referral) and simultaneously provide the detailed SNP identification and reporting that are (or will be) required to initiate a patient-specific treatment regimen.

The M. tuberculosis detection and genotyping concordance data indicate that the amplification microarray consumable and test are reproducible and are equally efficacious when primary sputum or decontaminated sediments are used as the test input. When one considers that the automated workstation consistently extracted more M. tuberculosis DNA from primary sputum than from the paired sediment and that smear-negative sputum extracts were more likely than sediment extracts to give a strong output of RIF or INH resistance (Table S1), one could argue that primary sputum is the preferred sample type for the TruTip/amplification microarray test.

The clinical samples for this study were intentionally selected to estimate amplification microarray genotyping specificity. With only 74 DST results and four phenotypically RIF-resistant samples, however, there are not enough data to draw conclusions about amplification microarray genotyping sensitivity. We do know that the INH-reporting probes used here provide limited coverage of INH resistance-conferring mutations (48, 49); the power of an amplification microarray is the ability to increase the number of INH resistance-conferring genes and SNPs into the test (e.g., see reference 12).

### Summary.

This study integrated a complex MDR-TB microarray work flow into an entirely closed-amplicon, integrated microfluidic device (the amplification microarray) and demonstrated its efficacy and substantial equivalence to other methods for M. tuberculosis detection and genotyping for RIF and INH resistance with primary sputum and decontaminated sediments. The microfluidic simplification, shelf life, and analytical performance of the amplification microarray are well aligned with the consensus product profiles for new M. tuberculosis diagnostics and represent a significant ease-of-use advantage over other (manual) hybridization-based tests for MDR-TB. We are now able to expand the test coverage for additional resistance-conferring mutations, integrate primers and probes that are predictive of an XDR phenotype (6, 11), and combine the TruTip and amplification microarray technologies into a sample-to-answer system.

## Supplementary Material

Supplemental material
