# Supplementary material for: Genotyping Multidrug-Resistant Mycobacterium tuberculosis from Primary Sputum and Decontaminated Sediment with an Integrated Microfluidic Amplification Microarray Test
Source: J Clin Microbiol. 2018 Feb 22;56(3):e01652-17. doi: 10.1128/JCM.01652-17 (PMC5824040; doi:10.1128/JCM.01652-17)
Supplement: Supplemental material [file supp_56_3_e01652-17__index.html]

Supplemental material 

# Genotyping Multidrug-Resistant Mycobacterium tuberculosis from Primary Sputum and Decontaminated Sediment with an Integrated Microfluidic Amplification Microarray Test

## Supplemental material

- Supplemental file 1 -

  Table S1 (Itemized data for all clinical samples)

  XLSX, 23K
